# The inhibitory effect of berberine chloride hydrate on *Streptococcus mutans* biofilm formation at different pH values

**DOI:** 10.1128/spectrum.02170-23

**Published:** 2023-09-25

**Authors:** Yang Zhou, Zhuoying Liu, Jie Wen, Yan Zhou, Huancai Lin

**Affiliations:** 1 Hospital of Stomatology, Guanghua School of Stomatology, Sun Yat-Sen University, Guangzhou, China; 2 Guangdong Provincial Key Laboratory of Stomatology, Sun Yat-Sen University, Guangzhou, China; The Ohio State University, Columbus, Ohio, USA

**Keywords:** *Streptococcus mutans*, biofilm, berberine chloride hydrate, pH, virulence

## Abstract

*Streptococcus mutans* (*S. mutans*) is one of the major cariogenic bacteria of dental caries owing to its ability to adhere to tooth surfaces and biofilm formation. Berberine chloride hydrate (BH), a quaternary ammonium salt alkaloid, has diverse pharmacological efforts against microorganisms. However, the effect of BH on *S. mutans* biofilm has not been reported. Considering that berberine is a quaternary ammonium salt alkaloid, which needs to adapt to a large variation in pH values and the acid resistance of *S. mutans*, we employed three groups including pH 5 (acidic), pH 8 (alkaline), and unprocessed group (neutral) to examine the antibiofilm activities of BH against *S. mutans* during different pH values. In this study, we found BH effectively suppresses *S. mutans* biofilm formation as well as its cariogenic virulence including acid production and EPS synthesis significantly, and the inhibitory effort was reduced under acidic condition whereas elevated under alkaline condition. In addition, we preliminarily explored the influence of pH values on the structural stability and biosafety of BHas well as the underlying mechanism of inhibition of *S. mutans* biofilm formation with BH. Our study showed BH could maintain a good structural stability and low toxicity to erythrocytes at different pH values. And BH could downregulate the expression of *srtA, spaP,* and *gbpC,* which play critical roles in the adhesion process, promoting bacterial colonization and biofilm formation. Furthermore, *comX* and *ldh* expression levels were downregulated in BH-treated group, which might explain its inhibitory effect on acid production.

**IMPORTANCE:**

Dental caries is a common chronic detrimental disease, which could cause a series of oral problem including oral pain, difficulties in eating, and so on. Recently, many natural products have been considered as fundamental sources of therapeutic drugs to prevent caries. Berberine as a plant extract showed good antibiofilm abilities against microorganism. Our study focuses on its antibiofilm abilities against *S. mutans,* which was defined as major cariogenic bacterium and explored the role of pH values and possible underlying mechanisms in the inhibitory effect of BH on *S. mutans* biofilm formation. This study demonstrated a promising prospect for BH as an adjuvant drug in the prevention and management of dental caries.

## INTRODUCTION

Dental caries, a common chronic detrimental disease, results from the destruction of the mineralized dental hard tissues due to the complicated interactions between a cariogenic dental plaques, high-carbohydrate diet, host susceptibility, and other factors (1, 2). *Streptococcus mutans* (*S. mutans*) is a gram-positive endogenous bacterium, implicated as the major cariogenic bacterium (3, 4) and lives primarily in biofilms on the tooth surfaces (5). Within the biofilm, *S. mutans* utilizes sugars to synthesize exopolysaccharides (EPS) and metabolize carbohydrates into lactic acids, attacking tooth structure and causing tooth decay, leading to dental caries (4, 6). Furthermore, the cariogenic potential of *S. mutans* also relies on the ability to thrive under low pH conditions (5, 7). Biofilm is a structured cluster of different bacteria encapsulated in extracellular polymeric substances, which consist of extracellular polymeric substances, extracellular DNA (eDNA), lipoteichoic acids, and proteins, thereby creating a protective barrier to traditional drugs (8, 9). It has been shown that biofilm can exhibit more tolerance to antibiotics than their counterpart planktonic microorganisms, which could be up to 1,000 times (10, 11). Thus, controlling biofilm formation can be an effective method of caries prevention.

Current adopted strategies to solve the biofilm-associated problem were mainly through antibiotics and physical-mechanical techniques (such as high-velocity spray and jet irrigators) (12). However, although the antibiotics are usually effective in caries treatment, excessive use of antibiotics may lead to changes in oral and intestinal flora, as well as adverse reactions such as vomiting, diarrhea, and dental plaque (13). Meanwhile, due to the complexity and difficulty of the physical-mechanical approaches, despite advances in biofilm disruption and removal, most approaches still apply conventional antibiotic-based therapy (12). Thus, numerous natural products have been considered as fundamental sources of therapeutic agents or substitutes of conventional antibacterial drugs due to their reduced cytotoxicity and diminished possibility of resistance (14, 15).

**Fig 1:**
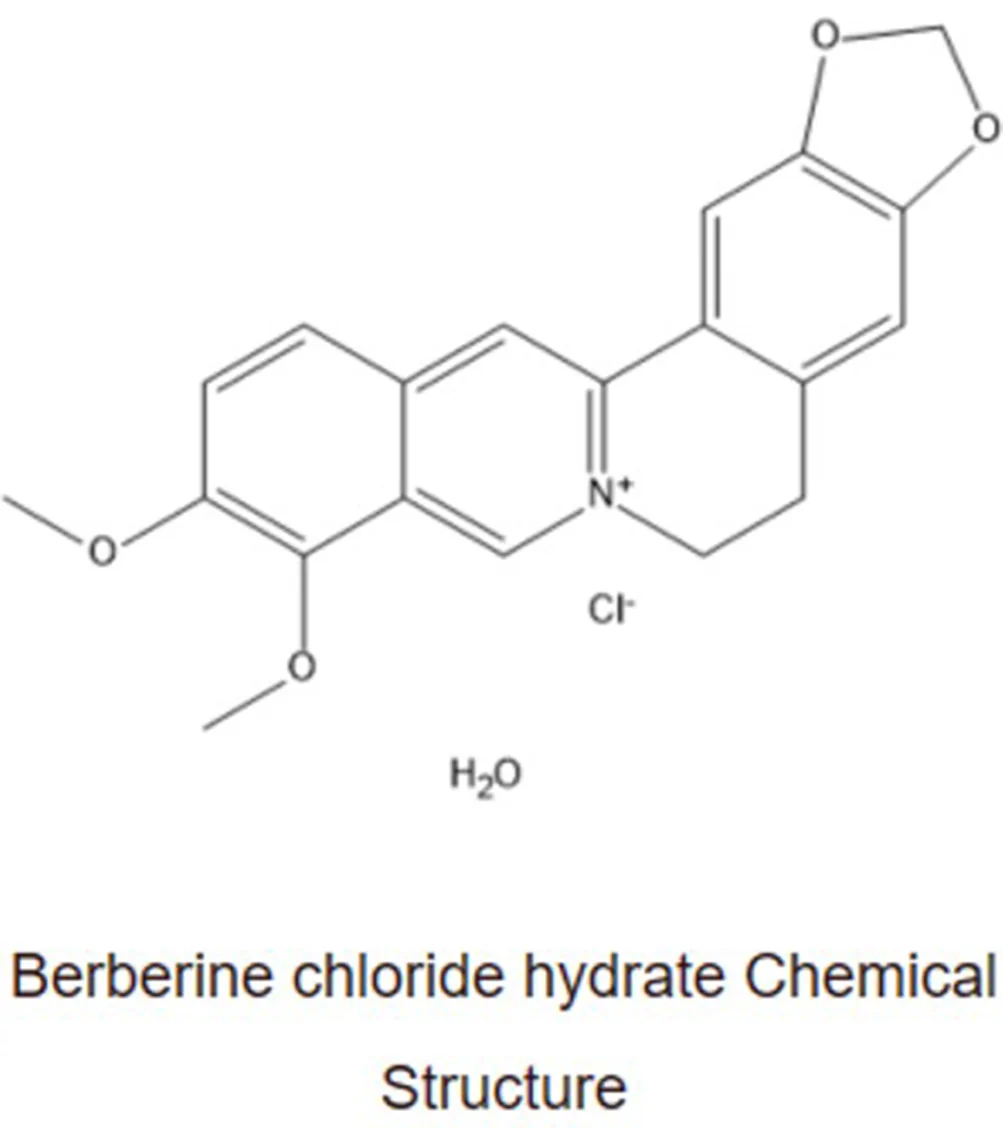
The molecular structure of berberine chloride hydrate (BH).

Berberine, a quaternary ammonium salt alkaloid with a long history in traditional Chinese medicine, can be isolated in abundance from medicinal plants (roots and rhizomes) such as *Hydrastis canadensis* (goldenseal), *Berberis vulgaris* (barberry), *Berberis aquifolium* (Oregon grape), and *Berberis aristata* (16 – 18). It has been used to treat various diseases, including cancer, diabetes, cardiovascular diseases, hyperlipidemia, and central nervous system disorders because of its anticancer, anti-inflammatory, and antimicrobial effects (19 – 21). Chloride or sulfate salt of berberine (Fig. 1) is commonly used for clinical purposes (17). Furthermore, berberine also possesses a significant antimicrobial effect on various microorganisms, including bacteria, viruses, and fungi (16). Recently, Arkadiusz Dziedzic et al. (16) found that berberine can effectively inhibit the growth of *S. mutans in vitro*. Maryam Kazemipoor et al. (22) reported that barberry plant extract and CHX (chlorhexidine) have similar antimicrobial activity against *S. mutans*. However, in our knowledge, the antibiofilm effect of berberine on *S. mutans* has received little or no attention.

In this study, considering that berberine is a quaternary ammonium salt alkaloid, which needs to adapt to a large variation in pH values (23) and the acid resistance of *S. mutans*, we investigated the antibiofilm activities of berberine chloride hydrate (BH) against *S. mutans* during different pH values and preliminarily explored the influence of pH values on the structural stability and biosafety of BH as well as the underlying mechanism of inhibition of *S. mutans* biofilm formation with BH. Our findings contribute to provide insight into the clinic uses of berberine as an antibiofilm agent, indicating its therapeutic potential of berberine in the prevention and management of dental caries.

## RESULTS

### Effect of BH on *S. mutans* biofilm formation, biofilm metabolic activity, and planktonic growth at different pH values

**Fig 2:**
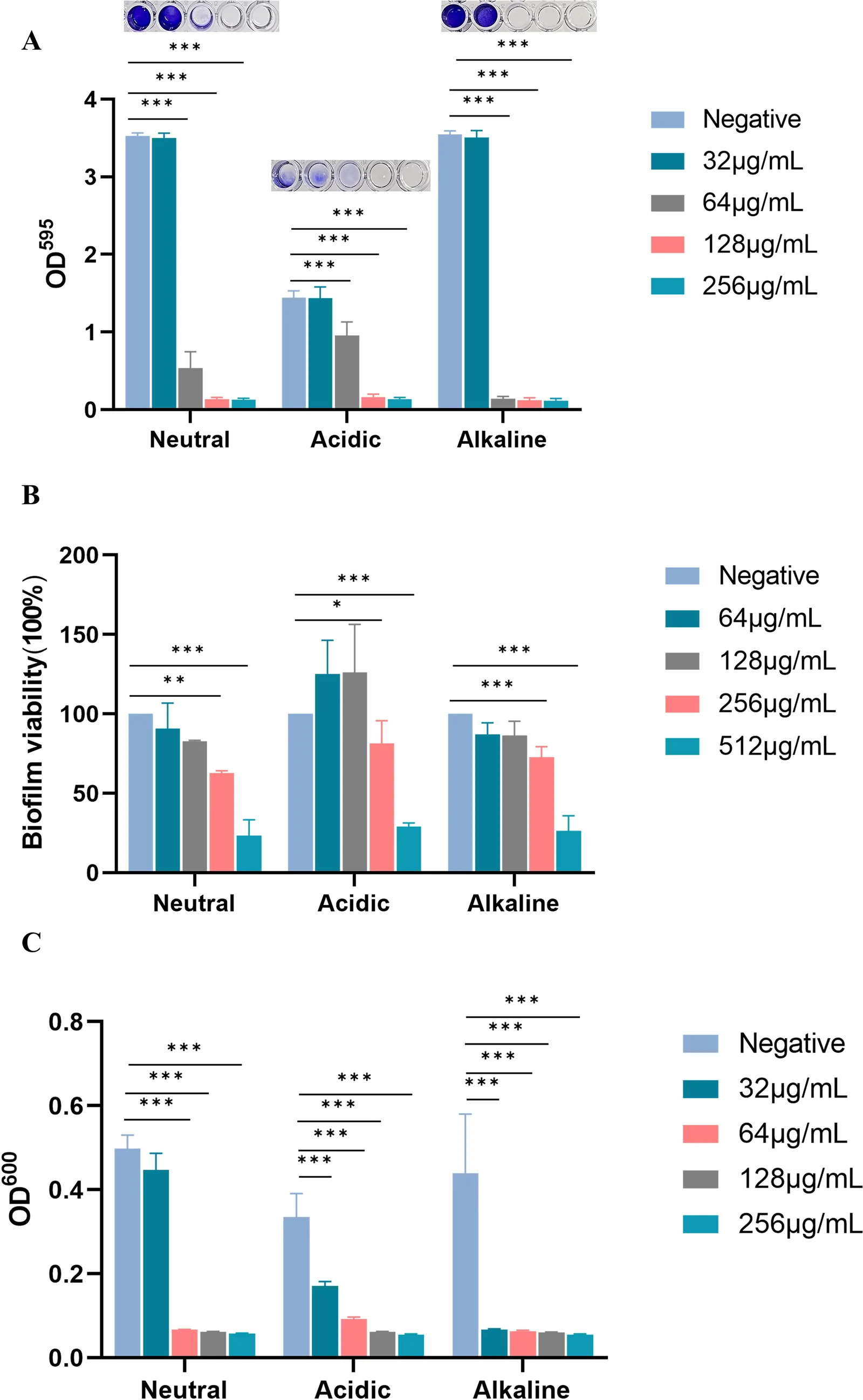
(**A) **Effect of BH on *S. mutans* biofilm formation at different pH values. (**B**) Effect of BH on *S. mutans* biofilm dispersion at different pH values. (**C**) Effect of BH on planktonic proliferation of *S. mutans* at different pH values. *P < 0.05, **P < 0.01, ***P < 0.001.

To clarify the effect against *S. mutans* biofilm formation, the crystal violet staining method was performed. As shown in Fig. 2A, when the concentration of BH reached 64 µg/mL, the biofilm biomass of *S. mutans* was obviously reduced in the neutral group than that of the negative group (*P* < 0.001). Whereas, for acidic group, BH could inhibit *S. mutans* biofilm formation above 64 µg/mL (*P* < 0.001) than that of negative group in which biofilm biomass was nearly half of the neutral negative group, but the decreased biofilm biomass was much lower than neutral group. For alkaline group, the biofilm biomass of *S. mutans* was significantly reduced at 64 µg/mL of BH than that of negative group (*P* < 0.001), yet the decreased biofilm biomass was higher than the neutral group.

**Fig 3:**
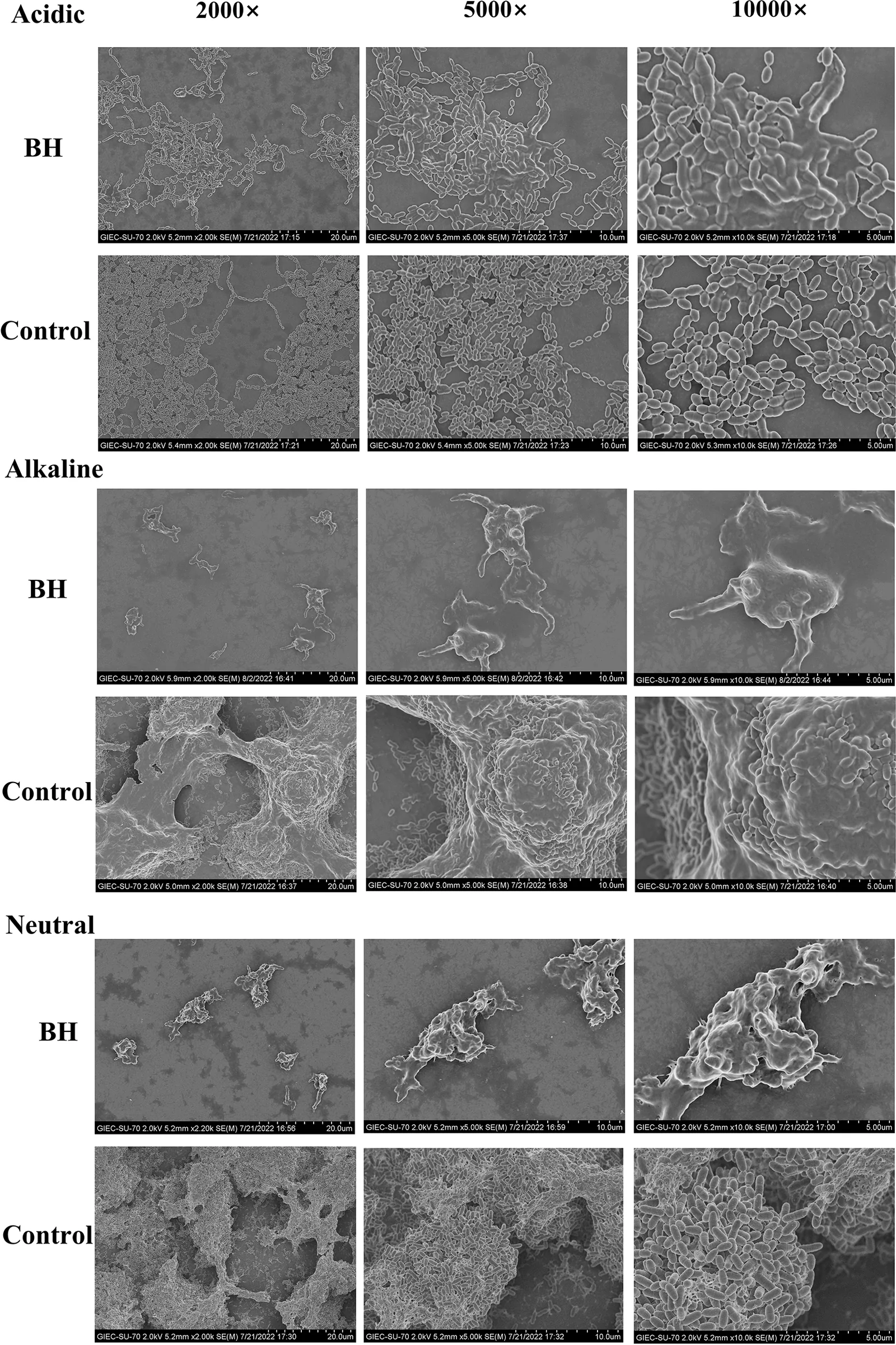
Morphological architectures of *S. mutans* biofilms in the presence of BH were visualized by SEM. Photos of *S. mutans* biofilms were taken via SEM at 2,000×, 5,000×, and 10,000× magnifications at different pH values.

The impact of BH on biofilm morphological changes was then evaluated by scanning electron microscopy (SEM) (Fig. 3). In this study, after treated with BH, the number of *S. mutans* cells in neutral group was obviously reduced compared with those of the control group, and alkaline group showed the same tendency. However, at acidic group, the bacterial cells displayed a dispersed short-chain distribution without being enveloped in a number of extracellular polysaccharides in the control group, whereas the BH-treated group showed no significant changes. It means that the biofilm formation of *S. mutans* could be inhibited in the acidic condition, and the inhibitory effect of BH on *S. mutans* biofilm formation was decreased under acidic condition.

In addition, we also tested the effect of BH on the viability of *S. mutans* cells in a mature biofilm. As shown in Fig. 2B, BH exhibited a dispersion effect against the preformed biofilm. When BH was at 256 µg/mL (*P* < 0.001), the metabolic ability of biofilm began to decrease compared with control group in the neutral group. Furthermore, acidic group and alkaline group exhibited the same trend as neutral group, both the concentration of BH-inhibiting biofilm metabolic ability was 256 µg/mL (*P* < 0.05). This indicated that the acidic or alkaline condition does not influence the inhibitory effort of bacterial cells within *S. mutans* matured biofilm.

We further tested the antibacterial effects of BH on planktonic *S. mutans*. Interestingly, we found that BH inhibited the growth of planktonic *S. mutans* at acidic group and alkaline group above the concentration of 32 µg/mL (*P* < 0.001), which were lower compared with that of neutral group (64 µg/mL) (Fig. 3C). This indicated that the antibacterial effect of BH was enhanced under acidic and alkaline condition.

### Inhibition of EPS synthesis and bacterial viability within *S. mutans* biofilms by BH at different pH values

**Fig 4:**
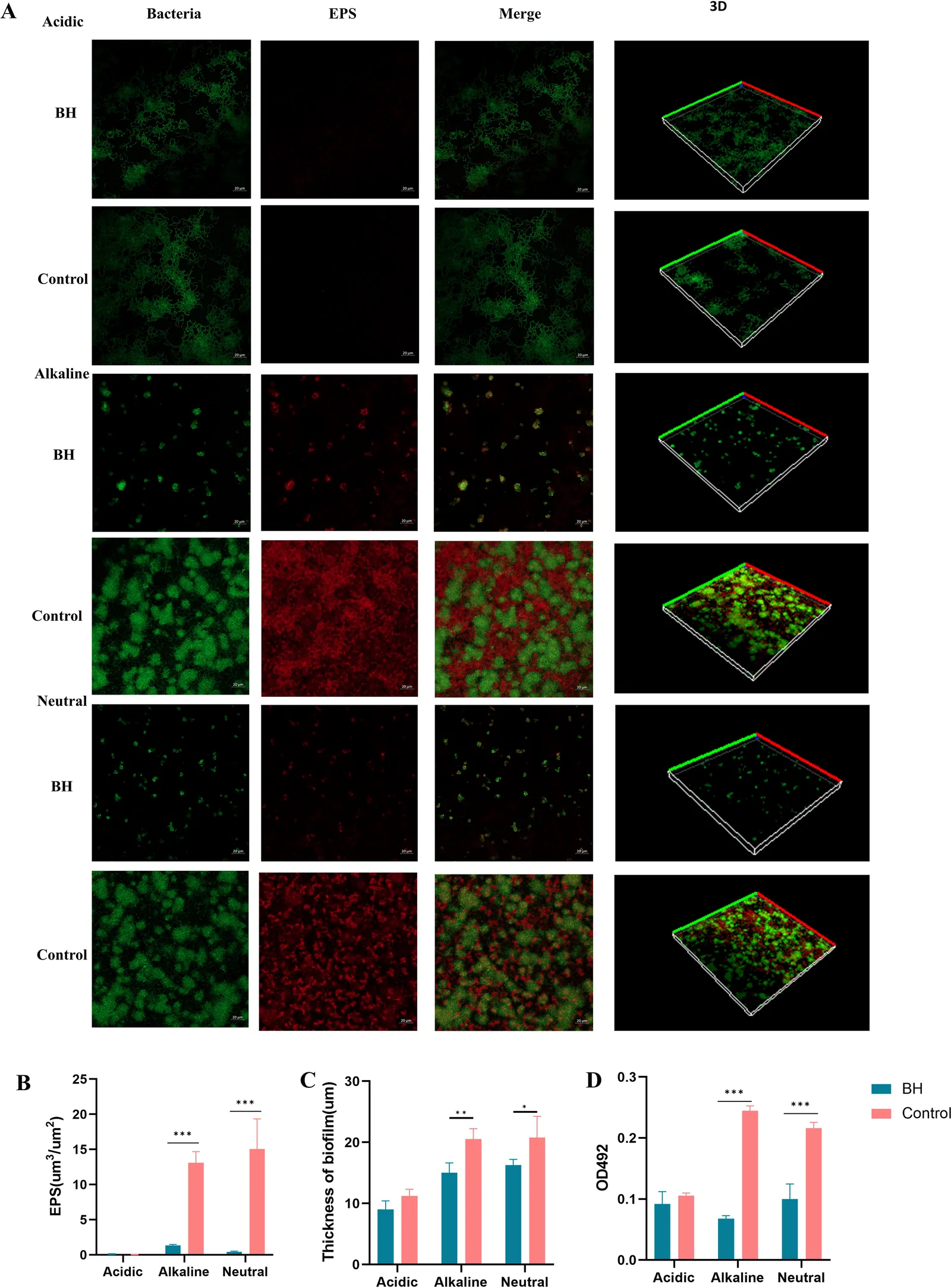
Effect of BH on the EPS synthesis of *S. mutans* biofilms at different pH values. (**A**) Representative CLSM images of *S. mutans* biofilms with or without BH at different pH values. Red fluorescence (647 labeled Dextran) identifies EPS, green fluorescence (SYTO9) identifies bacteria. (**B**) Quantification of EPS synthesis within biofilms. (**C**) The thickness of the biofilms. (**D**) Quantitative water-insoluble EPS analysis in biofilms. *P < 0.05, **P < 0.01, ***P < 0.001.

The development of *S. mutans* biofilm is a synergistic process of bacterial accumulation and EPS generation. Confocal laser scanning microscopy (CLSM) analysis of biofilms was applied to observe the effect of BH on biofilm architecture and EPS synthesis within *S. mutans* biofilms. We found, at neutral group and alkaline group, the biofilms treated with BH showed much less bacteria and polysaccharides than that of control group, which consequently resulted in an apparent reduction in EPS synthesis (Fig. 4A and B, *P* < 0.001, *P* < 0.001). Moreover, the thickness of the biofilms showed significant reduction than that of control group (Fig. 4C, *P* < 0.05, *P* < 0.001). However, for acidic group, the biofilms showed relatively loose structure hardly with polysaccharides, which resulted in thinner thickness in acidic control group, and no difference was observed between control group and BH-treated group (Fig. 4A through C).

Consistently, phenol/H2SO4 method was performed to detect water-insoluble glucan within biofilms. In general, BH-treated group synthesized remarkably less water-insoluble glucan than that of control group in alkaline group and neutral group, respectively. For acidic group, the control group exhibited less water-insoluble glucan than neutral control group, whereas no significant difference was found between the BH-treated group and control group (Fig. 4D, *P* < 0.001).

**Fig 5:**
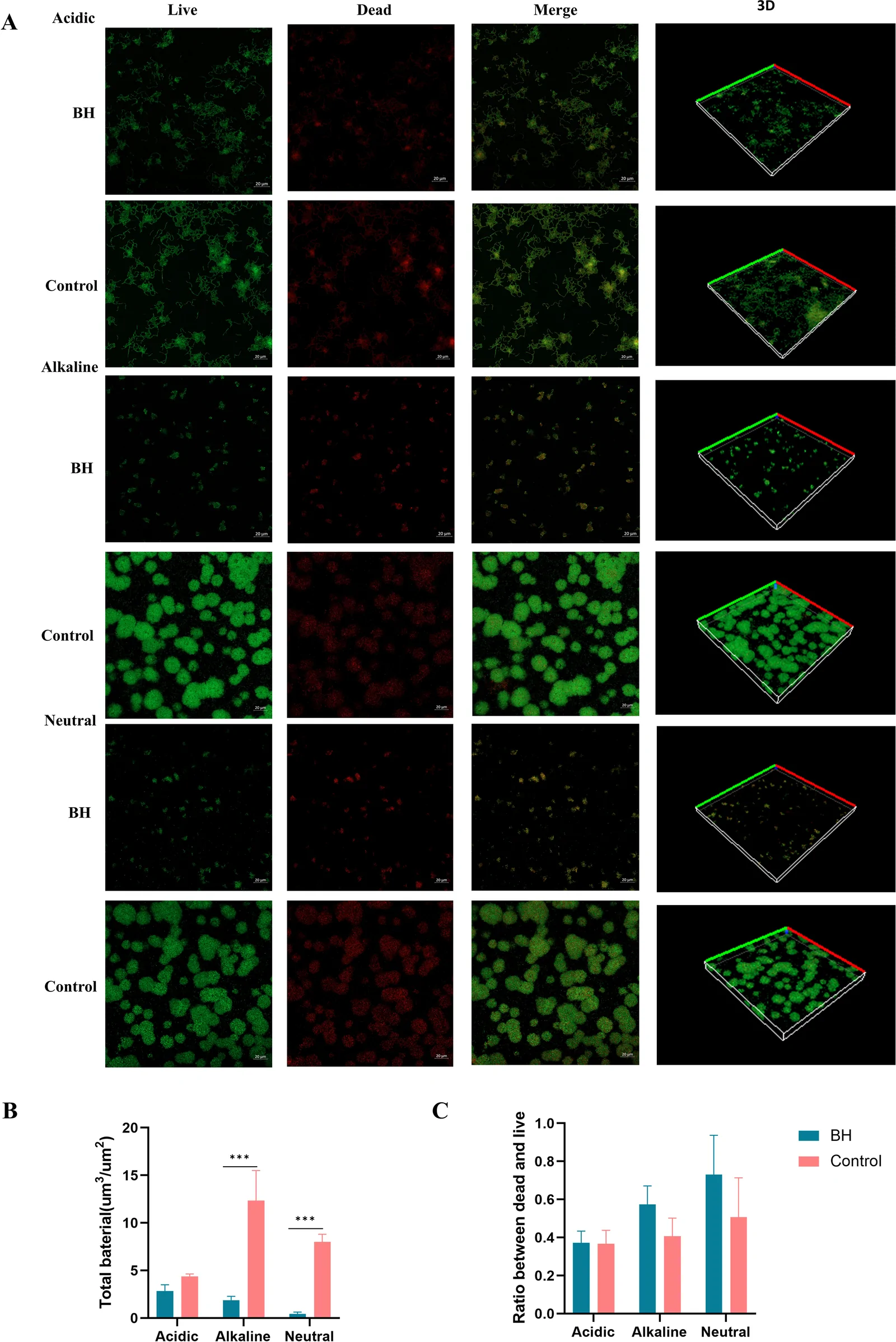
Effect of BH on *S. mutans* biofilms bacterial viability at different pH values. (**A**) Representative pictures of dead/live bacteria within the *S. mutans* biofilms with or without BH at different pH values. Red fluorescence (PI) identifies dead bacterial, green fluorescence (SYTO9) identifies live bacterial. (**B**) Quantitative analysis of total bacteria in biofilms. (**C**) The ratio of dead/live in the biofilms. *P < 0.05, **P < 0.01, ***P < 0.001.

To observe the bacterial effect of BH on biofilms, live/dead staining was managed. After treatment with BH, the live bacteria (Fig. 5A and C) and total bacteria (Fig. 5A and B, *P* < 0.001) in the alkaline group and neutral group were both decreased. For acidic group, the control group exhibited lower total bacteria and live bacteria than neutral control group, and there was no significant difference between the control and BH-treated group (Fig. 5A through C). Above all, these results confirmed that the reduction in EPS by BH is parallel to the removal of bacteria within the biofilms and the inhibitory effort could be suppressed in the acidic condition.

### Suppression of acid production within *S. mutans* biofilmby BH at different pH values

**Fig 6:**
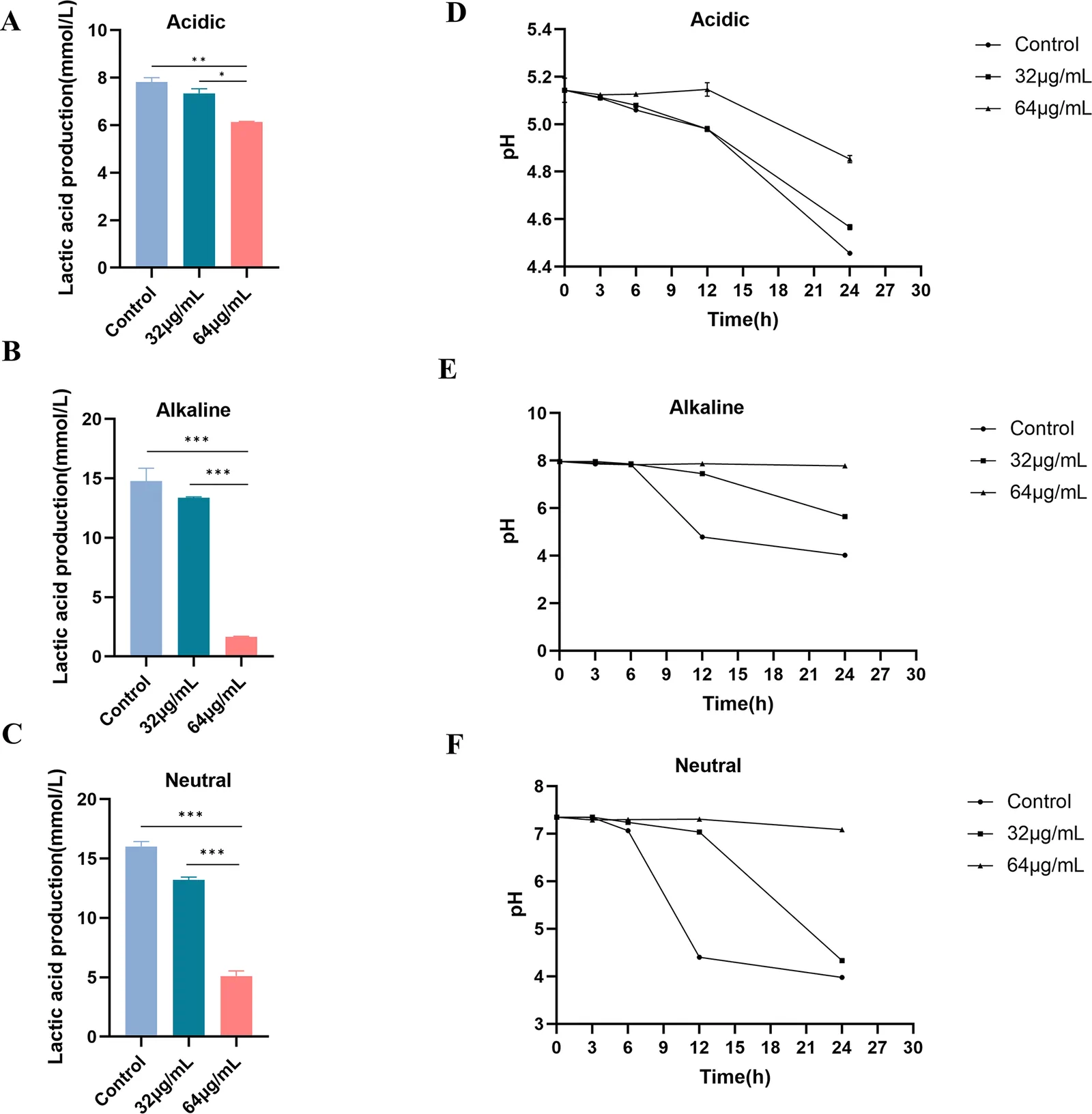
Acid production within *S. mutans* biofilms in response to treatment with BH at different pH values. (**A, B, and C**) pH of the culture mediums at acidic, alkaline, and neutral groups, respectively. (**D, E, and F**) Quantification of lactic acid production at acidic, alkaline, and neutral groups, respectively. *P < 0.05, **P < 0.01, ***P < 0.001.

Acid production is deemed as one of the major cariogenic virulence factors of *S. mutans.* We then used pH measurement and lactic acid measurement to observe the effect of BH on *S. mutans* acid production. As shown in Fig. 6A through C (*P* < 0.05, *P* < 0.01, *P* < 0.001), when treated with 64 µg/mL of BH, the lactic acid production was obviously reduced than that of the control group and BH (32 µg/mL) group among the acidic, alkaline, and neutral group. However, though BH could reduce the lactic acid production in acidic condition, the reduction was much lower than neutral and alkaline groups. Furthermore, the *in situ* pH of *S. mutans* biofilm at acidic (pH 5), alkaline (pH 8), and neutral group (pH 7.4) after 24 h were measured, respectively, and shown as 4.45, 4.02, and 3.98. This indicated that the metabolic activity of *S. mutans* was reduced at acidic condition. However, at neutral and alkaline groups, we observed the decrease in the pH (ΔpH) values for the biofilms treated with BH (64 µg/mL) were significantly lower than that of the control group (Fig. 6E and F). Interestingly, when the concentration of BH was at 32 µg/mL, BH can only slow the ΔpH values at alkaline group (Fig. 6E), which proves again the inhibitory effort of BH could be enhanced in alkaline condition. Nevertheless, at acidic group, after treatment with 64 µg/mL of BH, the ΔpH values were not significantly different from that of the control and BH (32 µg/mL) groups. (Fig. 6D). Taken together, decreased lactic acid production caused by BH could be inhibited in acidic condition and enhanced at alkaline condition.

### The structural stability and biosafety of BH at different pH values

Due to the effect of BH influenced by pH values, the structural stability and biosafety of BH at different pH values were measured using high-performance liquid chromatography (HPLC) analysis and hemolytic assay. As shown in Fig. S1, a narrow peak shape was found nearly at the same time at different pH values, which indicated that the structural stability of BH was not affected by pH values. What’s more, we found BH showed less damage to erythrocytes at different pH values, which suggest its biosafety (Fig. S2).

### Inhibition of expression of *S. mutans* virulence genes within biofilms by BH

**Fig 7:**
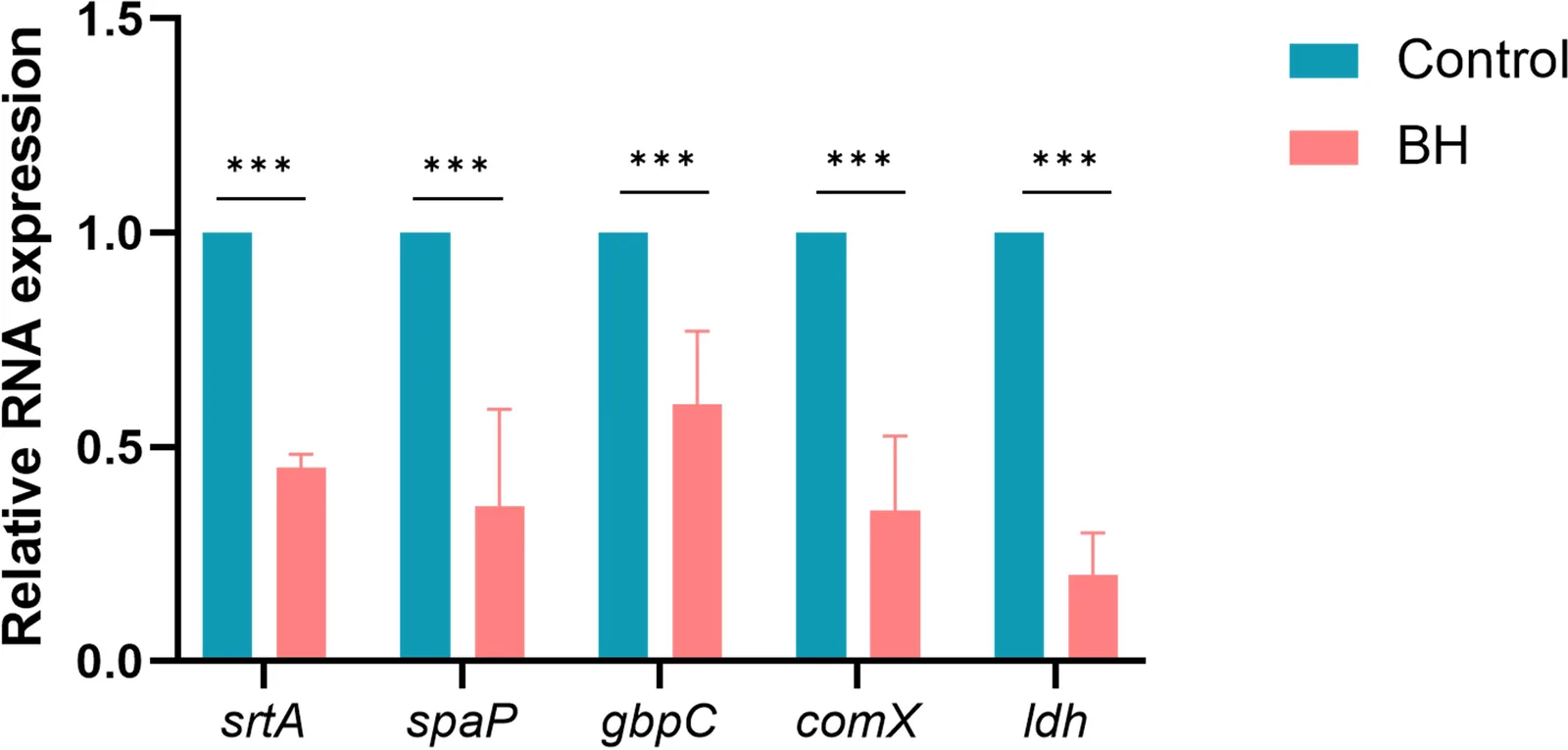
The mRNA expression levels of *srtA*, *spaP*, *gbpC*, *comX*, and *ldh* in bacterial cells within biofilms were quantified by RT-PCR. *P < 0.05, **P < 0.01, ***P < 0.001.

To further explore the regulatory effect of BH on *S. mutans* cells within biofilms, the expression levels of virulence genes involved in the biofilm formation were determined using RT-PCR. In our study, compared with control group, adhesion-associated gene *srtA* and *spaP* were significantly downregulated 0.55-fold and 0.64-fold, respectively (Fig. 7, *P* < 0.001), and quorum sensing (QS)-associated gene *comX* was significantly downregulated 0.65-fold than control group (Fig. 7, *P* < 0.001). GbpC encoded by gene *gbpC*, as the core regulators of *S. mutans* biofilm development, could regulate bacterial attachment to glucans and biofilm formation. Lactate dehydrogenase encoded by *ldh* is crucial for carbohydrate metabolism. Our study showed the expression levels of gene *gbpC* and *ldh* in the biofilms treated with BH were significantly downregulated 0.4-fold and 0.8-fold, respectively, when compared with the levels in control biofilms (Fig. 7, *P* < 0.001).

## DISCUSSION

*S. mutans* is the primary microorganism that adheres to tooth surfaces to create biofilm and is regarded as the most important pathogen involved in the development of dental caries (24, 25). Therefore, effective biofilm control has been identified as the key elements of dental caries management. BH, a chloride salt of berberine, was even demonstrated to inhibit oral microorganism biofilm formation, including that of *Candida albicans* (26), *Enterococcus faecalis* (21), and *Staphylococcus* spp. (27). However, the effect of BH on *S. mutans* biofilm has not been studied yet. As is known, *S. mutans* could thrive under environmental stress conditions, particularly low pH (28). We were curious about the effects of BH on *S. mutans* at different pH values. Thus, we employed three groups including pH 5 (acidic), pH 8 (alkaline), and unprocessed group (neutral) to observe the effects of BH on the biofilm formation of *S. mutans*.

In our study, BH exhibited good antibiofilm activity against *S. mutans* above the 64 µg/mL of BH under neutral condition, but the inhibitory effort was reduced in acidic condition while elevated in alkaline condition. This is similar to the previous study, which has been elucidated that the activity of tested quaternary ammonium salts approaches close to zero at pH 5 values and remains practically constant over the whole range of pH 7–10.5 (23). To determine whether BH prevented the biofilm formation related to its bactericidal effects, we further investigated its antibacterial action. We found that the growth of planktonic *S. mutans* could be suppressed induced by 32 µg/mL of BH under acidic and alkaline conditions, which was lower than the concentration to suppress biofilm formation. Therefore, we informed that the reduction in biofilm biomass may not be parallel to the elimination of bacterial activity. Furthermore, when BH was at 32 µg/mL under neutral condition, the growth of planktonic *S. mutans* was not influenced, indicating that the inhibitory effort of BH was elevated in acidic and alkaline conditions. However, it is not yet clear why berberine has an increased inhibitory effect on planktonic bacteria in acidic environment, indicating a potential area for future research.

Biofilm dispersion is also an important indicator for antibiofilm applications, which is one of the strategies used to prevent dental caries (29). In this study, we found BH could inhibit the performed biofilm metabolic activity above the concentration of 256 µg/mL. The dispersion effect of BH for performed biofilm has been reported in researches on different bacterial strains. Lihua Chen et al. found that BH exerts a dispersion effect on the formed biofilm of *Enterococcus faecalis* at a concentration of 800 µg/mL (21). A diverse repertoire of dispersion effect of BH was observed, might be attributed to the different strains, growth conditions, and detection techniques employed. In addition, we found the inhibitory effort for matured biofilm was not influenced by the pH values, which differs from biofilm formation, therefore further research was needed to study the effect of BH on the formed biofilm of *S. mutans* at different pH values.

Biofilms are composed primarily of bacterial cells and extracellular matrix. The most abundant component of extracellular matrix is EPS, accounting for 50–90% of the total volume of biofilms and can be considered as a main scaffold of biofilm (20, 30). In general, SEM is able to depict clearly the heterogeneity characteristic of biofilms. However, SEM may give a simplistic view of biofilms due to the dehydration process during sample preparation. Therefore, CLSM allows noninvasively observing the tridimensional structure and reactivity of biofilms, it was applied in our study (20, 31). Our SEM and CLSM results showed that BH could significantly reduce EPS production within *S. mutans* biofilm under neutral and alkaline conditions. Furthermore, our findings from live/dead bacterial staining indicated that the reduction in live and total bacterial cells within *S. mutans* biofilms after BH treatment further result in decreased EPS synthesis under neutral and alkaline conditions. Nevertheless, the EPS production and bacterial cells were obviously decreased in the acidic control group than neutral control group, along with the reduction of inhibitory effort of BH under acidic condition.

One of the major cariogenic virulence of *S. mutans* is the metabolic activity of acid production. The mineral composition of dental tissues is highly susceptible to increases in the levels of organic acid generated by carbohydrate metabolism. The pH started to fall as the lactic acid accumulated and dental demineralization occurs once pH falls below 5.5 (24, 32, 33). In our study, we found BH could inhibit the generation of lactic acid, thus preventing the acidification of the growth medium of *S. mutans* biofilm effectively under neutral and alkaline conditions, thereby preventing tooth demineralization. However, due to the weak reduction of acid production, BH could not effectively prevent demineralization of tooth tissue under acidic condition.

As discussed previously, BH could suppress biofilm formation, EPS synthesis, and acid production in *S. mutans,* and the inhibitory effort was reduced under acidic condition whereas elevated under alkaline condition. Thus, we employed HPLC analysis and hemolysis assay to preliminarily explore the influence of pH values on the structural stability and biosafety of BH. Our results showed that BH could maintain a good structural stability and low toxicity to erythrocytes at different pH values. Based on it, we supposed that the effect of BH that exerts an inhibitory effect on *S. mutans* biofilm formation may be influenced by different pH values, though the structure remains stable. However, the specific mechanism between BH and pH needs further study.

Then, we were curious about the regulatory network underlying these effects of BH on *S. mutans* biofilm formation. We thus studied the expression of several virulence factors during biofilm development. Another cariogenicity of *S. mutans* is adhesion, which can promote bacterial colonization and biofilm development (9). SrtA, encoded by *srtA* gene, aids in the insertion of surface proteins with a sorting signal into cell walls. SrtA catalyzed the surface-associated protein P1 (SpaP) of *S. mutans*, which is involved in the initial adhesion process and biofilm formation (4, 34). In addition, glucan-binding proteins (GbpCs), another surface-associated protein, mediate the bacterial attachment to glucans and biofilm formation of *S. mutans* (35, 36). In our study, we found the gene expression of *srtA*, *spaP,* and *gbpC* were significantly lower in the BH-treated group compared to that of control group, which might explain its inhibitory effect on biofilm formation. QS, which can sense the bacterial number, withstand acidic condition, and activate virulence factors, promotes initial adhesion and biofilm formation (37, 38). ComX, acts as a sigma factor, could directs the transcription of several late competence-specific genes (39). Our data showed BH could cause the downregulated expression of *comX,* these findings corroborate the report that *comX*-deficient mutants exhibit decreased biofilm biomass and lacked architectural integrity (40). Furthermore, lactate dehydrogenase, which is encoded by *ldh,* is an important factor involved in the cariogenic potential of *S. mutans* and contributes to the production of lactic acid in *S. mutans* (33). In the current study, we found the gene expression of *ldh* in BH-treated group was also significantly downregulated than control group, implying that the maintenance of the highest pH in the BH-treated group was caused by the low level of *ldh* expression.

In conclusion, our results demonstrated that BH can effectively suppress *S. mutans* biofilm formation as well as its cariogenic virulence including acid production and EPS synthesis, and the inhibitory effort was reduced under acidic condition whereas elevated under alkaline condition. To our knowledge, this is a new report regarding the effect of BH on *S. mutans* biofilm formation at different pH values. This finding demonstrated a potential for BH as an adjuvant therapeutic drug in the prevention and management of dental caries.

## MATERIALS AND METHODS

### Compound preparation, bacterial strain, and growth conditions

BH was purchased from MCE (Medchem Express, Shanghai, China), dissolved in distilled water to obtain the solution of 1024 µg/mL, and stored at −80°C before use. *S. mutans* UA159 (ATCC 700610) was cultured under anaerobic conditions (80% N_2_, 10% H_2_, and 10% CO_2_) at 37°C in a brain heart infusion (BHI; Difco, Detroit, MI, USA). The concentration of bacteria was adjusted to 1 × 10^6^ colony-forming units (CFU)/mL for the subsequent experiments once the mid-logarithmic growth stage reached. The biofilm formation was then investigated using BHI with 1% sucrose (BHIS). To adjust the pH of BHI and 1% BHIS broths to initial pH values of 8 (alkaline circumstances) and 5 (acidic conditions), NaOH and HCl were added. The pH value of unprocessed group (neutral condition) is 7.2.

### Planktonic growth assays

Overnight cultures of *S. mutans* with BHI broth under acidic, alkaline, and neutral conditions, which containing various concentrations of BH were incubated at 37°C anaerobically for 24 h. The vehicle of BH under acidic, alkaline, and neutral conditions was used as their counterpart negative control. The amount of planktonic microbe proliferation was detected with a spectrophotometer (Tecan, Reading, Switzerland) at 600 nm. All experiments were repeated three times.

### Biofilm formation assays

Crystal violet staining was utilized to examine the effect of BH on bacterial biofilm formation (41). Briefly, overnight cultures of *S. mutans* (1 × 10^6^ CFU/mL) with 1% BHIS under acidic, alkaline, and neutral conditions, which containing various concentrations of BH were inoculated anaerobically at 37°C for 24 h. The vehicle of BH under acidic, alkaline, and neutral conditions were used as their counterpart negative control. After 24 h, the media and unbound bacteria were discarded and PBS was used three times to rinse the wells. Then, methanol was added to mix the biofilms in the wells for 15 min. Following supernatant removal, the biofilms were dried for 30 min and then added 0.1% (wt/vol) crystal violet solution for 15 min, after which the staining area was rinsed gently with flowing water to remove the unbound dye. Subsequently, the dye was dissolved in 95% ethanol and shaken for 30 min at room temperature. Finally, the absorbance of dissolved crystal violet was measured at 595 nm.

### MTT metabolic assay

The metabolic activity of biofilm cells was evaluated by MTT assay with some modifications (9). In brief, overnight cultures of *S. mutans* (1 × 10^6^ CFU/mL) with 1% BHIS under acidic, alkaline, and neutral conditions were inoculated anaerobically at 37°C for 24 h to create mature biofilms. Following supernatants’ removal, 1% BHIS with various concentrations of BH was added to the plates. The vehicle of BH under acidic, alkaline, and neutral conditions was used as their counterpart negative control. Another 24 h later, the medium was discarded and thoroughly cleaned with PBS three times. Then the biofilms were added with 0.5 mg/mL of MTT solution and incubated at 37°C under dark for 1 h. Subsequently, the supernatants were discarded, and then added 100% DMSO to each well for 20 min under dark. Lastly, the absorbance of the mix solution was measured at 450 nm. The following formula was used to determine the rate of metabolic activity:


% Metabolic activity=[(OD450 nm of the BH treated group)/(OD450 nm of control group)]×100%

### SEM assay

The biofilm structure changes after treatment with BH were visualized using SEM (ZEISS, Oberkochen, Germany). Biofilms in the presence of BH (64 µg/mL) were formed in the six-well plate as described earlier. The vehicle of BH under acidic, alkaline, and neutral conditions were used as their counterpart negative control. After removing the medium, PBS was used to rinse the biofilms three times and 2.5% (wt/vol) glutaraldehyde was added for 6 h. Then the biofilms were washed three times with PBS and sequentially dehydrated with graded concentrations of ethanol (50%, 70%, 80%, 90%, 100% [vol/vol]) for 15 min. Following treatment, the biofilms were treated with a solution of *tert*-butanol three times (1 × 15 min), then dried overnight by lyophilization. Finally, the biofilm samples were sprayed with gold and observed by SEM at 2,000×, 5,000×, and 10,000× magnification.

### CLSM analysis

CLSM was applied for EPS staining and dead/live imaging. Biofilms in the presence of BH (64 µg/mL) were grown in 15 mm confocal dishes as described earlier. The vehicle of BH under acidic, alkaline, and neutral conditions were used as their counterpart negative control. For EPS staining, 2.5 µM Alexa Fluor 647-labeled (Invitrogen, USA) dextran conjugate was added to the dishes before the biofilm formation. After 24-h-old biofilm formation, the supernatant was removed and rinsed three times with distilled water, followed by incubated 2.5 µM SYTO9 green fluorescent dye (Invitrogen, USA) for 15 min under dark. For dead/live staining, after removal of the supernatant, the biofilms were washed with distilled water for three times and then stained with equal-amount mixture of SYTO9 (Invitrogen, USA) and propidium iodide (Invitrogen, USA) in accordance with the manufacturer’s instructions of the LIVE/DEAD BacLight Bacterial Viability Kit (L7012, Invitrogen, Carlsbad, CA, USA) for 15 min under dark. The excitation/emission for Alexa Fluor 647-labeled dextran conjugate and SYTO9 are 652/668 nm and 480/500 nm. And the excitation/emission for propidium iodide is 490/635 nm. Subsequently, LSM 980 (Zeiss, Oberkochen, Germany) was used to monitor the biofilms and capture the images at 20× immersion lens. The ImageJ COMSTAT software was used to analyze the images.

### Quantitative determination of water-insoluble EPS

Water-insoluble EPS within biofilms were examined using the phenol/H_2_SO_4_ method with some modifications (42). Biofilms in the presence of BH (64 µg/mL) were grown in the six-well plate as described earlier. The vehicle of BH under acidic, alkaline, and neutral conditions was used as their counterpart negative control. Following treatment, the excess medium in the well was discarded and rinsed twice with PBS. Then PBS was used to resuspend the precipitate in each well and centrifuged at 4,000 g for 10 min. After that, the supernatant was discarded and precipitate was then resuspended with NaOH (0.1 mol/L) and incubated at 37°C for 2 h. Thus, the water-insoluble glucans were contained in the supernatant produced by centrifugations. The EPSs were precipitated by filtering the supernatant through 0.22 mm nitrocellulose membrane filters, followed by added three volumes of chilled 95% ethanol and incubated overnight at 4°C. Subsequently, to create a red color, one volume of supernatant was then mixed with five volumes of concentrated sulfuric acid and one volume of ice cold 5% phenol. Lastly, the absorbance of each well was detected at 492 nm.

### Lactic acid and pH measurement

Lactic acid and pH measurement (1) were performed to detect acid production. Biofilms in the presence of BH (64 µg/mL) were grown in the 24-well plate as described earlier. The vehicle of BH under acidic, alkaline, and neutral conditions were used as their counterpart negative control. For pH measurement, the pH of the medium supernatants was measured with a pH electrode (Mettler Toledo, Zurich, Switzerland) at 3 h, 6 h, 12 h, and 24 h. For lactic acid measurement, biofilms were rinsed three times with PBS and then added buffered peptone water (BPW) composed of 0.2% sucrose to each well, followed by incubation at 37°C anaerobically for 2 h. Then the supernatants were examined using a lactate assay kit (catalog number A019-2; Jiancheng, Nanjing, China). The absorbance was monitored at 530 nm and the quantification of lactic acid was calculated using the following formula: lactic acid (mmol/L) = [(OD530 nm of the BH-treated group – OD530 nm of the blank group)/(OD530 nm of standard group – OD530 nm of the blank group)] * [(the concentration of standard group) (mmol/L)].

### HPLC analysis

The HPLC analysis of BH at different pH values was performed on an Agilent 1290 System with an Agilent Eclipse plusC18 column with the mobile phase consisting of eluent A (0.01% trifluoroacetic acid in water) and eluent B (0.05% trifluoroacetic acid in acetonitrile). The flow rate was set to 1.0 mL/min and the elution peak was measured at 265 nm.

### Hemolytic assay

Hemolytic assay (43) was applied to detect the toxicity to the erythrocytes with some modifications. The fresh blood from rabbits (catalog number E0503; Pingrui Biotech, Beijing, China) was placed into a 15 mL centrifuge tube. Then PBS was added to remove fibrous proteins, which were centrifuged at 1,500 rpm for 5 min. After three times, the blood cells were contained in the precipitate and then mixed with different concentrations of BH at different pH values. The distilled water was set as the positive control. Afterward, the samples were incubated in 37°C for 1 h and were subsequently centrifuged at 1,500 rpm for 5 min. Then the absorbance of supernatant was evaluated at 540 nm.

### RNA extraction and quantitative real-time PCR (qRT-PCR)

The qRT-PCR method was applied to evaluate the relative expression levels of *S. mutans* virulence genes linked to biofilm development. Biofilms in the presence of BH (64 µg/mL) were grown in the 6-well plate as described earlier. The vehicle of BH under acidic, alkaline, and neutral conditions was used as their counterpart negative control. After incubation for 24 h, the biofilms were harvested by centrifugation at 12,000 rpm for 5 min at 4°C and miRNeasy Mini Kit (QIAGEN GmbH, Hilden, Germany) was used to extract the total RNA. The reverse transcription of the total RNA was performed using a PrimeScript RT reagent kit (Takara Bio Inc., Otsu, Japan). The relative mRNA expressions of virulence genes were evaluated by the qRT-PCR method. Primers are listed in Table 1. The 16S rRNA expression was used as an internal control. Real-time PCR detection was performed using a LightCycler 96 (Roche, USA) instrument. Finally, the gene expression was normalized to the reference gene 16S rRNA using a 2−ΔΔCT method.

**TABLE 1:** Nucleotide sequence of primers used for qRT-PCR

Gene	Primer sequence (5’−3’)	
	Forward	Reverse
srtA(43)	GAAGCTTCCTGTAATTGGCG	TTCATCGTTCCAGCACCATA
spaP(43)	GACTTTGGTAATGGTTATGCATCAA	TTTGTATCAGCCGGATCAAGTG
gbpC(9)	TCTGGTTTTTCTGGCGGTGT	GTCAATGCTGATGGAACGCC
comX(9)	CGTCAGCAAGAAAGTCAGAAAC	ATACCGCCACTTGACAAACAG
16sRNA(43)	CTTACCAGGTCTTGACATCCCG	ACCCAACATCTCACGACACGAG

### Statistical analysis

All experiments were performed three times independently. All data were statistically evaluated using GraphPad Prism 9 (GraphPad Company, San Diego, CA, USA). One-way analysis of variance (ANOVA) was conducted, followed by Dunnett’s multiple-comparison test or Tukey’s multiple-comparison test. *P*-value < 0.05 was used to determine the significant differences among the groups.

## SUPPLEMENTAL MATERIAL

Supplemental figure legends spectrum.02170-23-s0001.docx Legends for Fig. S1 and S2.

Fig. S1 spectrum.02170-23-s0002.tif HPLC analysis of BH at acidic, alkaline, and neutral conditions.

Fig. S2 spectrum.02170-23-s0003.tif Hemolysis ratio of BH at different pH values.

ASM does not own the copyrights to Supplemental Material that may be linked to, or accessed through, an article. The authors have granted ASM a non-exclusive, world-wide license to publish the Supplemental Material files. Please contact the corresponding author directly for reuse.

## References

[1] Sun Y , Pan Y , Sun Y , Li M , Huang S , Qiu W , Tu H , Zhang K . 2019. Effects of norspermidine on dual-species biofilms composed of Streptococcus mutans and Streptococcus sanguinis. Biomed Res Int 2019:1950790. doi:10.1155/2019/1950790 31781595 PMC6874952

[2] Wang S , Wang Y , Wang Y , Duan Z , Ling Z , Wu W , Tong S , Wang H , Deng S . 2019. Theaflavin-3,3'-digallate suppresses biofilm formation, acid production, and acid tolerance in Streptococcus mutans by targeting virulence factors. Front Microbiol 10:1705. doi:10.3389/fmicb.2019.01705 31404326 PMC6676744

[3] Hu P , Huang P , Chen MW . 2013. Curcumin reduces Streptococcus mutans biofilm formation by inhibiting sortase A activity. Arch Oral Biol 58:1343–1348. doi:10.1016/j.archoralbio.2013.05.004 23778072

[4] Hu P , Lv B , Yang K , Lu Z , Ma J . 2021. Discovery of myricetin as an inhibitor against Streptococcus mutans and an anti-adhesion approach to biofilm formation. Int J Med Microbiol 311:151512. doi:10.1016/j.ijmm.2021.151512 33971542

[5] Lemos JA , Palmer SR , Zeng L , Wen ZT , Kajfasz JK , Freires IA , Abranches J , Brady LJ . 2019. The biology of Streptococcus mutans. Microbiol Spectr 7. doi:10.1128/microbiolspec.GPP3-0051-2018 PMC6615571 30657107

[6] Wu J , Jiang X , Yang Q , Zhang Y , Wang C , Huang R , McBain AJ . 2022. Inhibition of Streptococcus mutans biofilm formation by the joint action of oxyresveratrol and Lactobacillus casei. Appl Environ Microbiol 88:e0243621. doi:10.1128/aem.02436-21 35416682 PMC9088390

[7] Li J , Wu T , Peng W , Zhu Y . 2020. Effects of resveratrol on cariogenic virulence properties of Streptococcus mutans. BMC Microbiol 20:99. doi:10.1186/s12866-020-01761-3 32303183 PMC7165372

[8] Folliero V , Dell’Annunziata F , Roscetto E , Amato A , Gasparro R , Zannella C , Casolaro V , De Filippis A , Catania MR , Franci G , Galdiero M . 2022. Rhein: a novel antibacterial compound against Streptococcus mutans infection. Microbiol Res 261:127062. doi:10.1016/j.micres.2022.127062 35597077

[9] Sun Y , Jiang W , Zhang M , Zhang L , Shen Y , Huang S , Li M , Qiu W , Pan Y , Zhou L , Zhang K , Dias FJ . 2021. The inhibitory effects of ficin on Streptococcus mutans biofilm formation. Biomed Res Int 2021:6692328. doi:10.1155/2021/6692328 33860052 PMC8009705

[10] Haney EF , Trimble MJ , Cheng JT , Vallé Q , Hancock REW . 2018. Critical assessment of methods to quantify biofilm growth and evaluate antibiofilm activity of host defence peptides. Biomolecules 8:29. doi:10.3390/biom8020029 29883434 PMC6022921

[11] Li J , Chen D , Lin H . 2021. Antibiofilm peptides as a promising strategy: comparative research. Appl Microbiol Biotechnol 105:1647–1656. doi:10.1007/s00253-021-11103-6 33475795

[12] Koo H , Allan RN , Howlin RP , Stoodley P , Hall-Stoodley L . 2017. Targeting microbial biofilms: current and prospective therapeutic strategies. Nat Rev Microbiol 15:740–755. doi:10.1038/nrmicro.2017.99 28944770 PMC5685531

[13] Ramirez J , Guarner F , Bustos Fernandez L , Maruy A , Sdepanian VL , Cohen H . 2020. Antibiotics as major disruptors of gut microbiota. Front Cell Infect Microbiol 10:572912. doi:10.3389/fcimb.2020.572912 33330122 PMC7732679

[14] Jeon JG , Rosalen PL , Falsetta ML , Koo H . 2011. Natural products in caries research: current (limited) knowledge, challenges and future perspective. Caries Res 45:243–263. doi:10.1159/000327250 21576957 PMC3104868

[15] Vuong TV . 2021. Natural products and their derivatives with antibacterial, antioxidant and anticancer activities. Antibiotics 10:70. doi:10.3390/antibiotics10010070 33450907 PMC7828331

[16] Dziedzic A , Wojtyczka RD , Kubina R . 2015. Inhibition of oral streptococci growth induced by the complementary action of berberine chloride and antibacterial compounds. Molecules 20:13705–13724. doi:10.3390/molecules200813705 26225951 PMC6332409

[17] Kumar A , Chopra K , Mukherjee M , Pottabathini R , Dhull DK . 2015. Current knowledge and pharmacological profile of berberine: an update. Eur J Pharmacol 761:288–297. doi:10.1016/j.ejphar.2015.05.068 26092760

[18] Caliceti C , Franco P , Spinozzi S , Roda A , Cicero AFG . 2016. Berberine: new insights from pharmacological aspects to clinical evidences in the management of metabolic disorders. Curr Med Chem 23:1460–1476. doi:10.2174/0929867323666160411143314 27063256

[19] Zhang C , Li Z , Pan Q , Fan L , Pan T , Zhu F , Pan Q , Shan L , Zhao L . 2022. Berberine at sub-inhibitory concentration inhibits biofilm dispersal in Staphylococcus aureus. Microbiology 168. doi:10.1099/mic.0.001243 36178801

[20] Wang X , Yao X , Zhu Z , Tang T , Dai K , Sadovskaya I , Flahaut S , Jabbouri S . 2009. Effect of berberine on Staphylococcus epidermidis biofilm formation. Int J Antimicrob Agents 34:60–66. doi:10.1016/j.ijantimicag.2008.10.033 19157797

[21] Chen L , Bu Q , Xu H , Liu Y , She P , Tan R , Wu Y . 2016. The effect of berberine hydrochloride on Enterococcus faecalis biofilm formation and dispersion in vitro. Microbiol Res 186–187:44–51. doi:10.1016/j.micres.2016.03.003 27242142

[22] Kazemipoor M , Fadaei Tehrani P , Zandi H , Golvardi Yazdi R . 2021. Chemical composition and antibacterial activity of Berberis vulgaris (Barberry) against bacteria associated with caries. Clin Exp Dent Res 7:601–608. doi:10.1002/cre2.379 33325156 PMC8404507

[23] BERGMANN F , SHIMONI A . 1952. Quaternary ammonium salts as inhibitors of acetylcholine esterase. II. pH dependence of the inhibitory effects of quaternary ammonium salts, and the dissociation constant of the anionic site. Biochim Biophys Acta 9:473–477. doi:10.1016/0006-3002(52)90195-9 13032143

[24] Guan C , Che F , Zhou H , Li Y , Li Y , Chu J , McBain AJ . 2020. Effect of rubusoside, a natural sucrose substitute, on Streptococcus mutans biofilm cariogenic potential and virulence gene expression in vitro. Appl Environ Microbiol 86:e01012-20. doi:10.1128/AEM.01012-20 32503907 PMC7414950

[25] Selwitz RH , Ismail AI , Pitts NB . 2007. Dental caries. Lancet 369:51–59. doi:10.1016/S0140-6736(07)60031-2 17208642

[26] Huang X , Zheng M , Yi Y , Patel A , Song Z , Li Y . 2020. Inhibition of berberine hydrochloride on Candida albicans biofilm formation. Biotechnol Lett 42:2263–2269. doi:10.1007/s10529-020-02938-6 32557120

[27] Guo N , Zhao X , Li W , Shi C , Meng R , Liu Z , Yu L . 2015. The synergy of berberine chloride and totarol against Staphylococcus aureus grown in planktonic and biofilm cultures. J Med Microbiol 64:891–900. doi:10.1099/jmm.0.000106 26272283

[28] Takahashi N , Nyvad B . 2011. The role of bacteria in the caries process: ecological perspectives. J Dent Res 90:294–303. doi:10.1177/0022034510379602 20924061

[29] Serretiello E , Folliero V , Santella B , Giordano G , Santoro E , De Caro F , Pagliano P , Ferro M , Aliberti SM , Capunzo M , Galdiero M , Franci G , Boccia G . 2021. Trend of bacterial uropathogens and their susceptibility pattern: study of single academic high-volume center in Italy (2015-2019). Int J Microbiol 2021:5541706. doi:10.1155/2021/5541706 34035817 PMC8116166

[30] Flemming HC , Neu TR , Wozniak DJ . 2007. The EPS matrix: the "house of biofilm cells J Bacteriol 189:7945–7947. doi:10.1128/JB.00858-07 17675377 PMC2168682

[31] Xu H , Wang C , Liang Z , He L , Wu W . 2015. The structure and component characteristics of partial nitrification biofilms under autotrophic and heterotrophic conditions. Appl Microbiol Biotechnol 99:3673–3683. doi:10.1007/s00253-014-6300-8 25529313

[32] Touger-Decker R , van Loveren C . 2003. Sugars and dental caries. Am J Clin Nutr 78:881S–892S. doi:10.1093/ajcn/78.4.881S 14522753

[33] Wang Y , Wang X , Jiang W , Wang K , Luo J , Li W , Zhou X , Zhang L . 2018. Antimicrobial peptide GH12 suppresses cariogenic virulence factors of Streptococcus mutans J Oral Microbiol 10:1442089. doi:10.1080/20002297.2018.1442089 29503706 PMC5827641

[34] Durso SC , Vieira LM , Cruz JNS , Azevedo CS , Rodrigues PH , Simionato MRL . 2014. Sucrose substitutes affect the cariogenic potential of Streptococcus mutans biofilms. Caries Res 48:214–222. doi:10.1159/000354410 24481032

[35] Haas W , Banas JA . 2000. Ligand-binding properties of the carboxyl-terminal repeat domain of Streptococcus mutans glucan-binding protein A. J Bacteriol 182:728–733. doi:10.1128/JB.182.3.728-733.2000 10633107 PMC94336

[36] Huang R , Li M , Gregory RL . 2015. Nicotine promotes Streptococcus mutans extracellular polysaccharide synthesis, cell aggregation and overall lactate dehydrogenase activity. Arch Oral Biol 60:1083–1090. doi:10.1016/j.archoralbio.2015.04.011 25985036

[37] Suntharalingam P , Cvitkovitch DG . 2005. Quorum sensing in streptococcal biofilm formation. Trends Microbiol 13:3–6. doi:10.1016/j.tim.2004.11.009 15639624

[38] Lu J , Cheng L , Huang Y , Jiang Y , Chu CH , Peng X , Li M , Xu HHK , Zhou X , Ren B . 2019. Resumptive Streptococcus mutans persisters induced from dimethylaminododecyl methacrylate elevated the cariogenic virulence by up-regulating the quorum-sensing and VicRK pathway genes. Front Microbiol 10:3102. doi:10.3389/fmicb.2019.03102 32038546 PMC6985435

[39] Cvitkovitch DG , Li YH , Ellen RP . 2003. Quorum sensing and biofilm formation in streptococcal infections. J Clin Invest 112:1626–1632. doi:10.1172/JCI20430 14660736 PMC281653

[40] Li Y-H , Tang N , Aspiras MB , Lau PCY , Lee JH , Ellen RP , Cvitkovitch DG . 2002. A quorum-sensing signaling system essential for genetic competence in Streptococcus mutans is involved in biofilm formation. J Bacteriol 184:2699–2708. doi:10.1128/JB.184.10.2699-2708.2002 11976299 PMC135014

[41] Cui G , Li P , Wu R , Lin H . 2022. Streptococcus mutans membrane vesicles inhibit the biofilm formation of Streptococcus gordonii and Streptococcus sanguinis. AMB Express 12:154. doi:10.1186/s13568-022-01499-3 36508003 PMC9743899

[42] Liu Y , Xu Y , Song Q , Wang F , Sun L , Liu L , Yang X , Yi J , Bao Y , Ma H , Huang H , Yu C , Huang Y , Wu Y , Li Y . 2017. Anti-biofilm activities from bergenia crassifolia leaves against Streptococcus mutans. Front Microbiol 8. doi:10.3389/fmicb.2017.01738 PMC5601420 28955316

[43] Li K , Zhan W , Chen Y , Jha RK , Chen X . 2019. Docetaxel and doxorubicin codelivery by nanocarriers for synergistic treatment of prostate cancer. Front Pharmacol 10:1436. doi:10.3389/fphar.2019.01436 31920642 PMC6930690

