# Supplementary material for: The inhibitory effect of berberine chloride hydrate on Streptococcus mutans biofilm formation at different pH values
Source: Microbiol Spectr. 2023 Sep 25;11(5):e02170-23. doi: 10.1128/spectrum.02170-23 (PMC10580975; doi:10.1128/spectrum.02170-23)
Supplement: Supplemental figure legends — Legends for Fig. S1 and S2. [file spectrum.02170-23-s0001.docx]

**Supplemental figure legends:**

Supplementary Figure 1. The HPLC analysis of BH at acidic (A), alkaline (B) and neutral condition (C).

Supplementary Figure 2. The hemolysis ratio of BH at different pH values. * P < 0.05, ** P < 0.01, *** P < 0.001.
