# Supplementary figures and images for: The inhibitory effect of berberine chloride hydrate on Streptococcus mutans biofilm formation at different pH values
Source: Microbiol Spectr. 2023 Sep 25;11(5):e02170-23. doi: 10.1128/spectrum.02170-23 (PMC10580975; doi:10.1128/spectrum.02170-23)

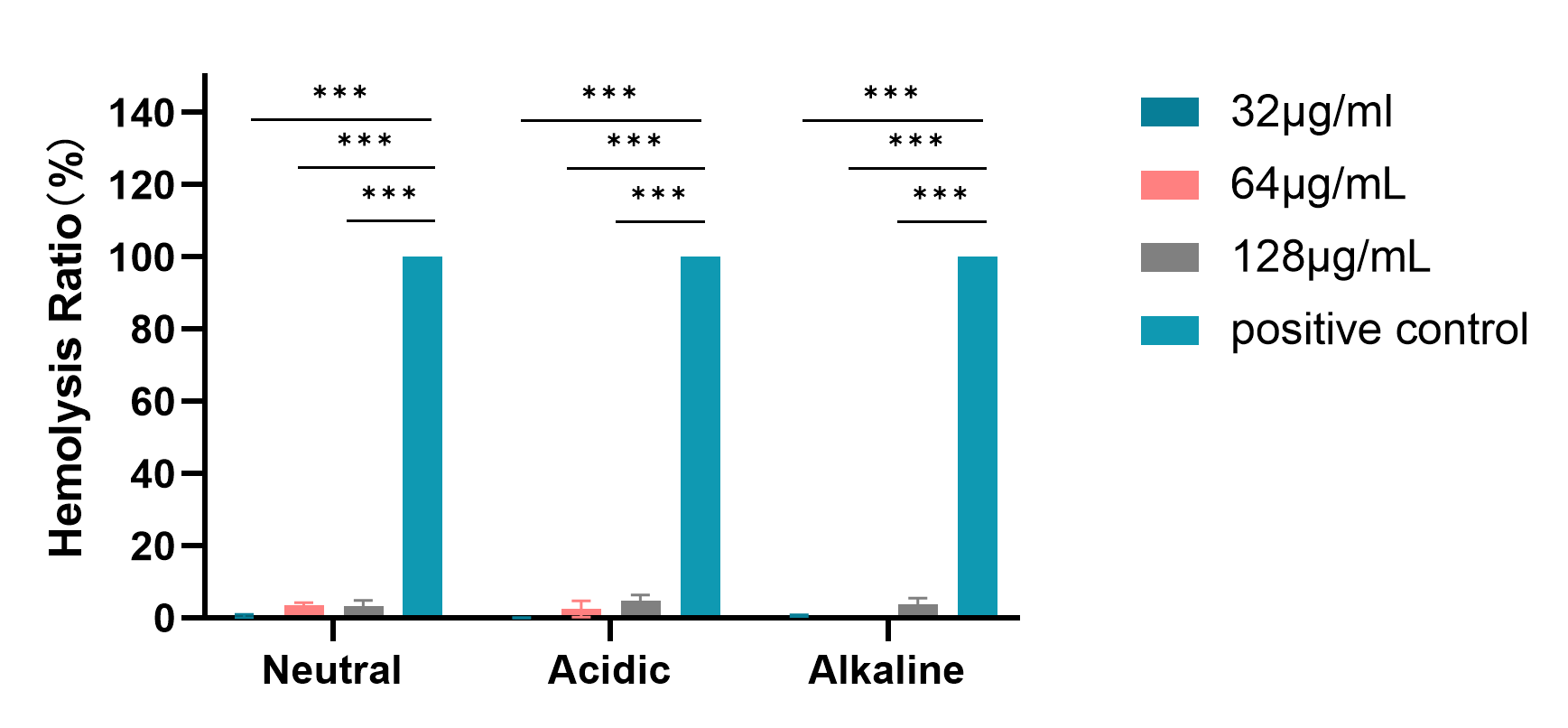

Supplement: Fig. S2 — Hemolysis ratio of BH at different pH values. [file spectrum.02170-23-s0003.tif]
